# RANKL deletion in periodontal ligament and bone lining cells blocks orthodontic tooth movement

**DOI:** 10.1038/s41368-017-0004-8

**Published:** 2018-02-26

**Authors:** Chia-Ying Yang, Hyeran Helen Jeon, Ahmed Alshabab, Yu Jin Lee, Chun-Hsi Chung, Dana T. Graves

**Affiliations:** 1 Department of Orthodontics, School of Dental Medicine, University of Pennsylvania, Philadelphia, PA USA; 2 Department of Periodontics, School of Dental Medicine, University of Pennsylvania, Philadelphia, PA USA

## Abstract

The bone remodeling process in response to orthodontic forces requires the activity of osteoclasts to allow teeth to move in the direction of the force applied. Receptor activator of nuclear factor-κB ligand (RANKL) is essential for this process although its cellular source in response to orthodontic forces has not been determined. Orthodontic tooth movement is considered to be an aseptic inflammatory process that is stimulated by leukocytes including T and B lymphocytes which are presumed to stimulate bone resorption. We determined whether periodontal ligament and bone lining cells were an essential source of RANKL by tamoxifen induced deletion of RANKL in which Cre recombinase was driven by a 3.2 kb reporter element of the Col1α1 gene in experimental mice (Col1α1.CreER^TM**+**^.RANKL^f/f^) and compared results with littermate controls (Col1α1.CreER^TM−^.RANKL^f/f^). By examination of Col1α1.CreER^TM+^.ROSA26 reporter mice we showed tissue specificity of tamoxifen induced Cre recombinase predominantly in the periodontal ligament and bone lining cells. Surprisingly we found that most of the orthodontic tooth movement and formation of osteoclasts was blocked in the experimental mice, which also had a reduced periodontal ligament space. Thus, we demonstrate for the first time that RANKL produced by periodontal ligament and bone lining cells provide the major driving force for tooth movement and osteoclastogenesis in response to orthodontic forces.

## Orthodontics: The mediators of bone remodeling

Cells and tissues have been identified that produce a signaling molecule essential for bone remodeling in orthodontic patients. Directional mechanical force, when applied to teeth, instigates changes to remodel the bone around the tooth. Researchers from the University of Pennsylvania School of Dental Medicine in Philadelphia, led by Dana Graves, genetically altered mice to remove ‘RANKL’, a protein thought to be a mediator in the breakdown of bone. The team found that the immune cells responsible for producing RANKL are primarily found in the tissue connecting tooth roots to the ridges of bone containing the tooth sockets and the protective cells that line the bones. Removing RANKL impaired the ability of the teeth to move in response to external directional force. This study provides insights into the relationship between RANKL production and orthodontic tooth movement.

## Introduction

Bone remodeling is essential for bone turnover and occurs mainly as a result of osteoclast and osteoblast activity^1,2^. The coordinated action of these two cell types leads to bone resorption and deposition, in response to stress and mechanical loading^3^. During orthodontic tooth movement, mechanical force is placed on a tooth so that it can be moved through the alveolar bone^4^. It is contingent upon the underlying cellular and molecular responses within the periodontal ligament (PDL) to an applied force. Osteoclasts are responsible for bone resorption and are derived from hematopoietic precursor cells of monocyte/macrophage lineage^5^. It has been reported that the receptor activator of nuclear factor-κB ligand (RANKL) and macrophage colony-stimulating factor (M-CSF) are essential for osteoclast formation^6^. RANKL binds to the receptor activator of nuclear factor-κB (RANK) and further initiates osteoclast formation and differentiation^7^. Multiple cell types have been reported to be capable of producing RANKL including both T and B lymphocytes, osteoblast precursors, mature osteoblasts, osteocytes, keratinocytes, mammary epithelial cells, vascular endothelial cells, synovial fibroblasts, cells within periodontal tissue, and hypertrophic chondrocytes^8^. During orthodontic tooth movement the transduction of mechanical forces to the cells triggers a biologic response, which has been described as an aseptic inflammation because it is mediated by a variety of inflammatory cytokines and does not represent a pathological condition^9,10^. The expression of inflammatory mediators after orthodontic force application is transitory and essential for orthodontic movement^11^. Therefore, leukocytes including T and B cells have been thought to be the major source of RANKL in response to the orthodontic forces.

Periodontal ligament, which connects the cementum of tooth to the alveolar bone, is a layer of mechanosensitive fibrous soft tissue that transduces the mechanic loading from tooth to alveolar bone^12,13^ and thus provides necessary microenvironment for cells participating in the alveolar bone remodeling^14^. Clinically, ''ankylosed teeth”, which lack PDL, cannot be moved by orthodontic force^15^. Therefore, PDL cells are supposed to play a pivotal role in osteoclast and osteoblast formation during orthodontic tooth movement. It is believed that the heterogeneous cells residing in PDL, including osteoblasts, osteoclasts, fibroblasts, cementoblasts, and progenitor/stem cells, release the cytokines which regulate this bone remodeling process^16^. These cytokines can recruit the osteoclast precursors from the bone marrow to the PDL space via blood stream. These osteoclast precursors differentiate into mature osteoclasts, which are further activated and resorb the alveolar bone under the pressure during the orthodontic tooth movement. To date, most of previous studies investigating on the response of PDL cells to orthodontic force are in vitro studies and focused on the relationship between PDL fibroblasts and the initiation of osteogenesis under mechanical force^17–19^ while few examined the mechanism between PDL fibroblasts and stimulation of osteoclastogenesis.

Transgenic mouse models are ideal to delineate the molecular actions of specific genes^20^. The ability to induce genetic recombination that lead to gene deletion through Cre recombinase adds another layer of analysis, particularly when Cre recombinase is controlled by a promoter element that restricts expression. The latter may facilitate lineage-specific gene deletion to well defined cell types. The addition of an inducible nuclear targeting domain that can be stimulated by application of an exogenous factor such as tamoxifen allows gene deletion to be controlled in a time-specific manner to address limitations of global constitutive germ-line deletion^21^. We examined transgenic mice in which loxP sites flanked exons 2–3 of RANKL and expressed Cre recombinase that had a nuclear targeting domain from a modified estrogen receptor that is activated by 4-hydroxytamoxifen (tamoxifen)^22^. It is reported that a tamoxifen inducible 3.2 kb collagen1α1 promoter element (Col1α1.CreER^TM^) is expressed in bone lining cells and osteoblasts at the early stages of differentiation and also in tendon and fascia fibroblasts but not skin fibroblasts^23,24^. The transgenic mice we examined had tamoxifen induced RANKL deletion mediated by the same 3.2 kb Col1α1 promoter controlled CreER^TM^ recombinase. The aim of our study was to determine whether PDL and bone lining cells are important sources of RANKL in orthodontic tooth movement by subjecting the first molar of genetically modified Col1α1.CreER^TM+^.RANKL^f/f^ and matched control mice to orthodontic forces.

## Results

### Animal status

At the start, the average ages were (14.1 ± 0.6) weeks (experimental Col1α1.CreER^TM+^.RANKL^f/f^ mice) and (14.4 ± 0.7) weeks (control Col1α1.CreER^TM−^.RANKL^f/f^ mice). The average weight is 21.63 g ± 3.51 g for the experimental Col1α1.CreER^TM+^.RANKL^f/f^ mice and 21.29 g ± 2.08 g for control Col1α1.CreER^TM−^.RANKL^f/f^ mice. After 12 days on delivery of the orthodontic appliance the average weight loss were 3.4 g ± 1.7 g for the experimental Col1α1.CreER^TM+^.RANKL^f/f^ mice and 3.2 g ± 1.1 g for control Col1α1.CreER^TM−^.RANKL^f/f^ mice. There was no significant difference in the amount of weight loss between experimental and control mice. In day 5 group we excluded one Col1α1.CreER^TM−^.RANKL^f/f^ mice due to the technical problem in tissue processing. In day 12 group we excluded one Col1α1.CreER^TM−^.RANKL^f/f^ mice due to possible abscess formation.

### Tissue specificity of Col1α1.CreER^TM^ induced by tamoxifen

**Fig. 1:**
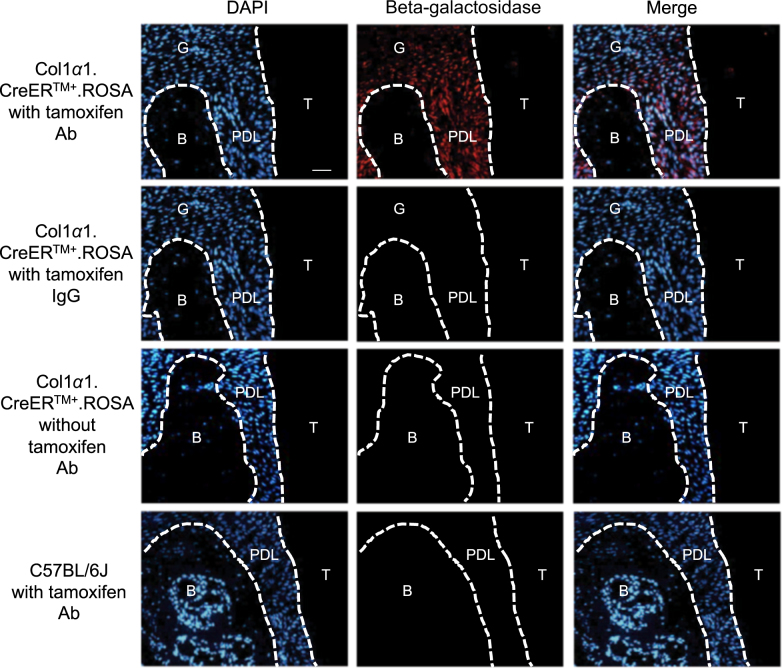
Beta-galactosidase expression in Col1α1.CreER+.ROSA26 reporter mice (x20). Beta-galactosidase was measured by immunofluorescence with specific antibody in sections from experimental mice with or without tamoxifen application or C57BL/6J control mice. Bar = 50 μm. Ab, beta-galactosidase antibody; IgG, control IgG; B, alveolar bone; G, gingiva; PDL, periodontal ligament; T, tooth. Original magnification 200x

**Fig. 2:**
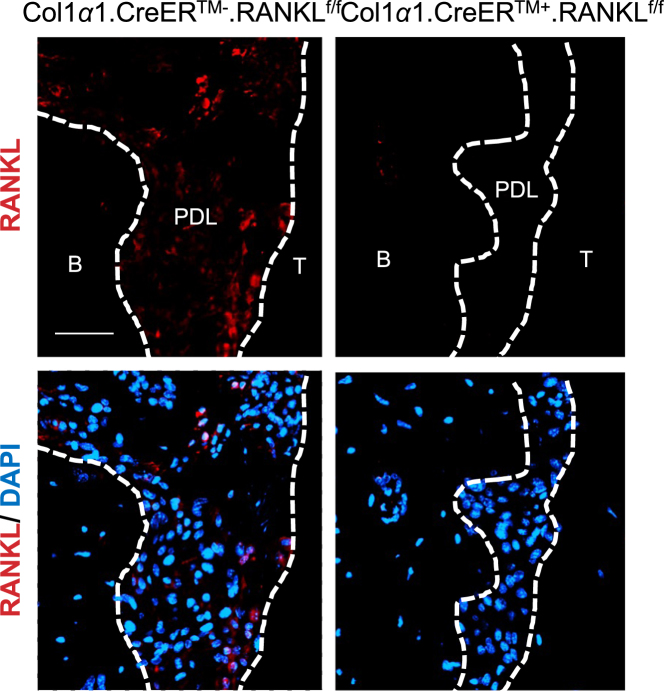
RANKL expression is ablated in PDL and bone lining cells in Col1α1.CreER+RANKLf/f experimental mice. RANKL expression was examined by immunofluorescence with antibody specific in sections from experimental and control mice. Bar = 50 μm. B, alveolar bone; PDL, periodontal ligament; RANKL, nuclear factor-κB ligand; T, tooth

Beta-galactosidase immunofluorescence of Col1α1.CreER.ROSA26 reporter mice revealed the tissue specificity of Col1α1.CreER^TM^ mice. Immunopositive cells were concentrated in the PDL and bone lining cells and consisted largely of fibroblastic cells as indicated by cells with a fusiform nucleus or a round nucleus with a tail. In addition, we found some immunopositive cells in the gingiva. The control groups which did not receive tamoxifen or did receive tamoxifen but were Cre negative showed no expression of the ROSA26 reporter (Fig. 1). RANKL expression in PDL of the experimental Col1α1.CreER^TM+^.RANKL^f/f^ mice was substantially reduced compared with control mice demonstrating lineage-specific deletion in PDL and bone lining cells (Fig. 2).

### Movement of teeth is reduced in experimental mice after orthodontic force application

**Fig. 3:**
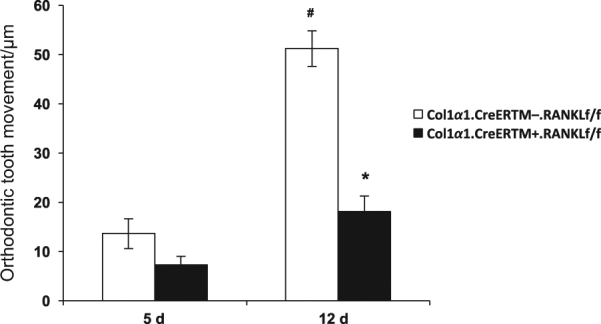
RANKL deletion in PDL and bone lining cells reduces orthodontic tooth movement. Orthodontic tooth movement was measured by micro-CT in sagittal images as described in figure 1. PDL, periodontal ligament; RANKL, nuclear factor-κB ligand. Each in value is the mean ± SEM for n=8 mice per group. *P < 0.05 versus Col1α1. CreER^TM−^.RANKL^f/f^ mice. #*P* < 0.05 versus day 5 Col1α1.CreER^TM−^.RANKL^f/f^ mice

Orthodontic tooth movement was measured by assessing the minimum distance between the distal of the first molar crown and the mesial of the second molar crown. After 5 days of mechanical loading, teeth in the control group (Col1α1.CreER^TM−^.RANKL^f/f^) had moved 14 µm and on day 12 moved 51 µm (*P* < 0.05, Fig. 3). At the 5 day time-point the amount of tooth movement decreased by 47% in the experimental Col1α1.CreER^TM+^.RANKL^f/f^ mice compared to the control Col1α1.CreER^TM−^.RANKL^f/f^ mice (*P* > 0.05). On day 12 the amount of orthodontic tooth movement was reduced by 65% in the experimental mice, which was statistically significant (*P* < 0.05). In the unloaded control side the distance between the teeth was zero or close to zero and there was no difference between groups (data not shown).

### PDL width is reduced in experimental mice after orthodontic force application

**Fig. 4:**
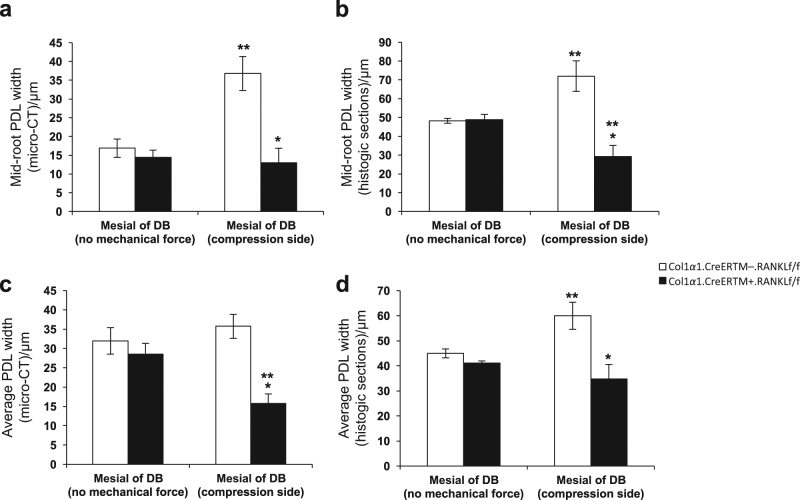
RANKL deletion in PDL and bone lining cells results in reduced PDL width during orthodontic tooth movement. PDL width was measured in micro-CT images as described in figure 1 or in histologic sections. PDL, periodontal ligament; RANKL, nuclear factor-κB ligand. Each value is the mean ± SEM for n=8 mice per group. *P < 0.05 versus Col1α1.CreER^TM−^.RANKL^f/f^ mice group. **P < 0.05 versus no mechanical force in control Col1α1.CreER^TM−^.RANKL^f/f^ mice

The effect of orthodontic forces was also examined by measuring the PDL width with several approaches by micro-CT or by histomorphometric analysis in TRAP stained sections from day 12 specimens. We assessed the PDL width on compression side, which is highly influenced by osteoclast resorption in response to orthodontic forces and compared it to PDL width in the contralateral side which did not receive orthodontic forces. The PDL width was measured at a midpoint in a buccal-palatal direction to obtain consistent results. It is our impression that the histologic measurements were more precise than micro-CT measurements because of the higher degree of resolution. In all of the groups there was no difference between experimental and wild type mice in the absence of orthodontic forces (*P* > 0.05). When measured at the midpoint of the root between the alveolar bone crest and root apex in sagittal views the application of orthodontic forces in wild-type mice causes an increase in PDL width suggesting that osteoclast activity at 12 days compensates for the compressive force applied (Fig. 4a, b). In comparison to no orthodontic forces, the application of a compressive force increased the PDL width at mid root in the wild-type group by 118% when analyzed by micro-CT and by 49% when analyzed in histologic sections (*P* < 0.05). In contrast there was no increase on the compression side in the experimental Col1α1.CreER^TM+^.RANKL^f/f^ mice. After 12 days of mechanical force the width of the PDL at the midpoint in the control Col1α1.CreER^TM−^.RANKL^f/f^ mice was 2.8-fold greater by micro-CT and 2.5-fold when measured histomorphometrically compared with the experimental Col1α1.CreER^TM+^.RANKL^f/f^ mice (*P* < 0.05). Similar results were obtained when the average PDL width was assessed although the magnitude of the differences was slightly less most likely due to the impact of tipping that affects the average PDL width more than it affects the width at the midpoint mark (Fig. 4c, d).

### Osteoclast formation is reduced in experimental mice after orthodontic force application

**Fig. 5:**
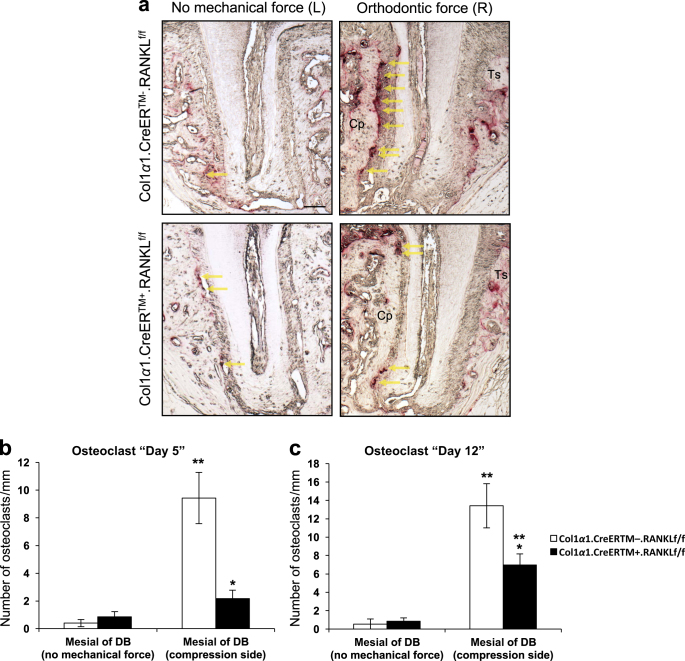
RANKL deletion in PDL and bone lining cells decreases osteoclast numbers in response to orthodontic forces. Osteoclasts were measured as bone lining, multi-nucleated, TRAP+ cells. **a** Representative images of TRAP stained sections (100x original magnification, distobuccal root). Bar, 100 μm. Cp, compression side; Ts: tension side. Yellow arrows indicate osteoclasts. **b** and **c** Quantification of osteoclasts on day 5 and 12. Each value is the mean ± SEM for n=8 mice per group. *P < 0.05 versus Col1α1.CreER^TM−^.RANKL^f/f^ mice. **P < 0.05 versus no mechanical force in control Col1α1.CreER^TM−^.RANKL^f/f^ mice. PDL, periodontal ligament; RANKL, nuclear factor-κB ligand; DB, distobuccal root

Bone resorption at the compression side is considered to be the rate-limiting step in orthodontic tooth movement^25^. In order to assess the effect of force on osteoclastogenesis and the impact of deleting RANKL in PDL fibroblasts, TRAP stained sections were examined to measure osteoclast numbers in bone mesial to the distobuccal root (compression side). In the absence of orthodontic forces there were relatively few osteoclasts. The application of orthodontic forces induced a large increase in the number of osteoclasts in the control mice and relatively few in the experimental mice (Fig. 5a). At 5 days there was a 20-fold increase in osteoclasts in the wild-type mice, which was maintained at 12 days (*P* < 0.05, Fig. 5b, c). On day 5 the osteoclast number was reduced by 77% in the experimental Col1α1.CreER^TM+^.RANKL^f/f^ mice compared to the control mice (*P* < 0.05, Fig. 5b). On day 12 the number of osteoclasts in Col1α1.CreER^TM+^.RANKL^f/f^ mice was reduced by 48% (*P* < 0.05, Fig. 5c).

## Discussion

In our study we found that deletion of RANKL through Cre recombinase driven by an inducible 3.2 kb promoter element of the Col1α1 promoter substantially reduced tooth movement. This was mechanistically explained by reduced osteoclast formation and failure to form a widened PDL in response to compressive forces.

Orthodontic tooth movement involves the complex interaction of several differentiated populations of cell types within the PDL^26^. The PDL contains leukocytes, neural, vascular and fibroblastic cells, many of which are thought to be capable of differentiating into osteoblasts or cementoblasts^16^. The PDL fibroblasts are spindle-shaped and elongated connective tissue cells that are located in the periodontal ligament^27^. They are the most abundant cells which occupy about 35% of the volume of the periodontal ligament space in rodent molars (blood vessels excluded)^28^ and are derived from less differentiated mesenchymal cells^29^. PDL fibroblasts are different from other fibroblasts because of their function in the formation and maintenance of the PDL in addition to their ability to differentiate to osteoblasts or cementoblasts and thereby contribute to repair, remodeling and regeneration of the adjacent alveolar bone and cementum^30^. They also exhibit characteristics of osteoblasts by showing high basal alkaline phosphatase (ALP) activity^31,32^. Moreover, PDL fibroblasts may participate in the osteoclasts differentiation through their cell–cell interactions with osteoclast progenitor cells^27^.

PDL fibroblasts are considered to be mechanosensitive cells of the PDL that are able to transduce mechanical strain into intracellular signals to surrounding cells and initiate remodeling of periodontal tissue including mineralized and non-mineralized tissue^33^. The 3.2 kb Col1α1 promoter that we used in this study is known to be highly active in immature pre-osteoblasts and fibroblasts within periodontal ligament^24^. By examining Col1α1.CreER.ROSA26 reporter mice we found the tamoxifen-induced Cre recombinase expression predominantly in the PDL fibroblasts. Thus, the reporter experiments indicate that tamoxifen induces the 3.2 kb Col1α1 promoter element and would lead to deletion of RANKL in PDL and bone lining cells. Based on our results we conclude that RANKL produced by these cells provides the major driving force for osteoclastogenesis due to the fact that the majority of tooth movement and osteoclastogenesis was removed in the experimental mice. This is logical because these cells are particularly sensitive to changes in mechanical forces. The 3.2 kb collagen 1α1 promoter element (Col1α1.CreER^TM^) that we examined induces Cre recombinase in bone lining cells, as well as tendon and fascia fibroblasts but not skin fibroblasts, leukocytes or endothelial cells^23,24^. By using transgenic mice that had RANKL deletion in PDL and bone lining cells we have identified these cells of mesenchymal origin as playing a major role in stimulating osteoclast formation through the production of RANKL in response to orthodontic forces.

Previous studies suggest that inflammation-mediated bone resorption can be attributed to the biological actions of B cells^34,35^ and T cells^36,37^. It has also been shown that peripheral release of vasoactive neurotransmitter after mechanical force triggers the migration of leukocytes into the capillaries and also activates various types of PDL cells^38^. Orthodontic tooth movement is known as an aseptic inflammation and it has been suggested that T and B cells play important roles in bone resorption during this movement. Our results indicate that PDL and bone lining cells are essential sources of RANKL but we did not examine leukocyte-specific gene deletion and cannot rule out the possibility that cells of hematopoietic origin are also a source of RANKL.

Other studies have examined the response of PDL cells to mechanical force but have largely been conducted in vitro and some of them have shown the capability of PDL cells to produce RANKL under compressive forces^39^. Human studies have found increased RANKL and decreased OPG expression in gingival crevicular fluid in humans after mechanical force although the cellular source of RANKL was not identified^40^. Kanzaki et al. discovered that RANKL expression is induced in compressed periodontal ligament cells and that this promotes osteoclastogenesis on the compression side in orthodontic tooth movement^41^. RANKL gene transfer significantly enhanced RANKL expression and tooth movement while inhibition of RANKL reduced tooth movement^41,42^.

Different animal models of orthodontic tooth movement have been described including rats, dogs, monkeys and mice^43^. The availability of genetically modified mice has provided valuable opportunities to discover the cellular or molecular mechanism involved in orthodontic tooth movement. However, the methodologies including the amount of applied force have varied. In some studies they placed an elastic band between the maxillary right first and second molars, in which the force magnitude was unknown^44^. Silva et al. proposed a standardized mouse model using a NiTi open coil spring bonded on the occlusal surface of maxillary first molar to the maxillary incisors with the exact force of 0.35 N, measured by a force gauge^45^. In this method bonding on the occlusal surface may cause occlusal interference that might induce occlusal trauma or increase the possible dislodgement of the orthodontic appliance. Yadav et al. used a NiTi closed coil spring with 0.004-inch SS ligature wires fixed at the CEJ of maxillary right first molar and the maxillary incisors, applying approximately 10 g of force to the subjected tooth^46^, which we used in our study. Weakness of this method is that the ligature wire tied around the CEJ might cause localized periodontal inflammation, adding a potential complicating factor.

RANKL is a key factor in stimulating osteoclastogenesis^47^. However, RANKL does not function alone as there are several other mediators that are also necessary to induce osteoclast formation which function in conjunction with RANKL or independently. M-CSF is essential for the formation of osteoclast precursors that are sensitized to RANKL stimulation through the induced expression of its cognate receptor, RANK. Prostaglandins, interleukin (IL)-17, IL-1, and other cytokines stimulate bone resorption by inducing RANKL expression. RANKL-independent osteoclastogenesis has been described in which several cytokines are able to substitute for RANKL including a proliferation-inducing ligand (APRIL), B cell-activating factor belong to the TNF family (BAFF), nerve growth factor (NGF), insulin-like growth factors (IGF) I, and IGF II^48^. Kim et al. reported that stromal cell–derived factor 1 (SDF-1) alone induces osteoclast differentiation from monocytes in the absence of RANKL^49^. Our studies reinforce the concept that RANKL is critical in orthodontic tooth movement since its deletion results in a significant blockage of force-induced osteoclast formation.

In conclusion, PDL and bone lining cells highly express Cre recombinase under the control of a 3.2 kb collagen 1α1 promoter element. The deletion of RANKL in PDL and bone lining cells greatly reduces osteoclastogenesis in response to mechanical force and prevents tooth movement indicating that these cells and RANKL are critical in response to orthodontic forces. These studies provide new insight into the cellular control of orthodontic tooth movement and provide evidence that the capacity to stimulate RANKL production by mesenchymal cells in the PDL may accelerate orthodontic treatment or inhibition of its expression may enhance resistance to orthodontic relapse.

## Materials and methods

### Animal model

Experiments were approved by the University of Pennsylvania Institutional Animal Care and Use Committee (Protocol #: 805011) and all methods were performed in accordance with guidelines and regulations relevant to the study. Mice expressing Cre recombinase under control of a 3.2 kb collagen 1α1 promoter element (Col1α1.CreER^TM^) were generously provided by Drs. Jian Feng and Henry Kronenberg as previously described^50^. Mice with floxed RANKL were generously provided by Dr. Charles O’Brien as described^51^. Both mice were backcrossed onto a C57BL/6 background. Transgenic mice were generated by crossing 3.2 kb Col1α1.CreER^TM**+**^ mice with RANKL floxed mice to generate experimental (Col1α1.CreER^TM+^.RANKL^f/f^) and control (Col1α1.CreER^TM−^.RANKL^f/f^) mice by Dr. Jian Q. Feng (Texas A&M University College of Dentistry, Dallas, TX). Sixteen Col1α1.CreER^TM+^.RANKL^f/f^ mice (10 males, 6 females) and sixteen Col1α1.CreER^TM−^.RANKL^f/f^ mice (6 males, 10 females) were randomly distributed into two groups examined after 5 and 12 days (*n* = 8 for each group). To induce Cre activity, we administered tamoxifen (5 mg per mouse per day, dissolved in 50 μL peanut oil) orally to Col1α1.CreER^TM+^.RANKL^f/f^ mice and Col1α1.CreER^TM−^.RANKL^f/f^ mice for 5 consecutive days as described^52^. To assess Cre recombinase expression we generated Col1α1.CreER.ROSA26 mice by crossing 3.2 kb Col1α1.CreER^TM^ mice with ROSA26 reporter mice from Jackson Laboratories (Bar Harbor, ME). We randomly divided Col1α1.CreER.ROSA26 mice into groups treated with tamoxifen or vehicle alone (*n* = 3 each). As another control we gave tamoxifen to the C57BL/6J mice (*n* = 3). Two to five mice were housed per cage under standard conditions with a 14-h light/10-h dark cycle. All the animals were closely monitored and fed a diet of powdered food, DietGel (ClearH_2_O, Westbrook, ME) and water ad libitum throughout the experimental period.

### Application of orthodontic force

Orthodontic appliance was delivered 1 week after the last administration of tamoxifen^53^. All the mice were 14 weeks old on delivery of the orthodontic appliance. Mice were anaesthetized by i.p. administration of ketamine (80 mg•kg^−1^), xylazine (5 mg⋅kg^−1^), and acepromazine (1 mg•kg^−1^). The maxillary and mandibular incisors were retracted with a customized mouth-opening device fabricated with 0.030-inch stainless steel (SS) wire. A custom-made 0.006” x 0.030” nickel–titanium closed-coil spring (Ultimate Wireforms, Inc., Bristol, CT) was used to deliver orthodontic force. The force/deflection rate (F/D) for the spring is known to be 10–12 g over a range of 0.5–1.5 mm activation^26^. This is the well-established method for the orthodontic tooth movement.^26,46,54^ In addition, we confirmed the force generated by 1–1.5 mm activation of the NiTi springs with the use of a tension gauge (data not shown). Under a dissecting microscope, a 4.5 mm NiTi closed coil spring was fixated between the maxillary right first molar and both of maxillary incisors with 0.004-inch SS ligature wires (Fort Wayne Metals, Fort Wayne, IN) connecting the coil spring to the teeth as previously described^26^. Two segments of 0.004-inch SS ligature wires were tied at the ends of the nickel-titanium coil spring. Ligature wire on the distal end was threaded through the contact between the first and second right maxillary molars and fixed around the cementoenamel junction (CEJ) of the maxillary right first molar. The wire on the mesial end of the spring was ligated around the both incisors and bonded in place with light-cured dental adhesive resin (Transbond XT; 3M Unitek, Monrovia, CA). No reactivation of the NiTi coil spring was performed during the entire experimental period. The contra-lateral left side served as the control. Mice were euthanized on day 5 and 12 after orthodontic force application.

### Micro-CT

**Fig. 6:**
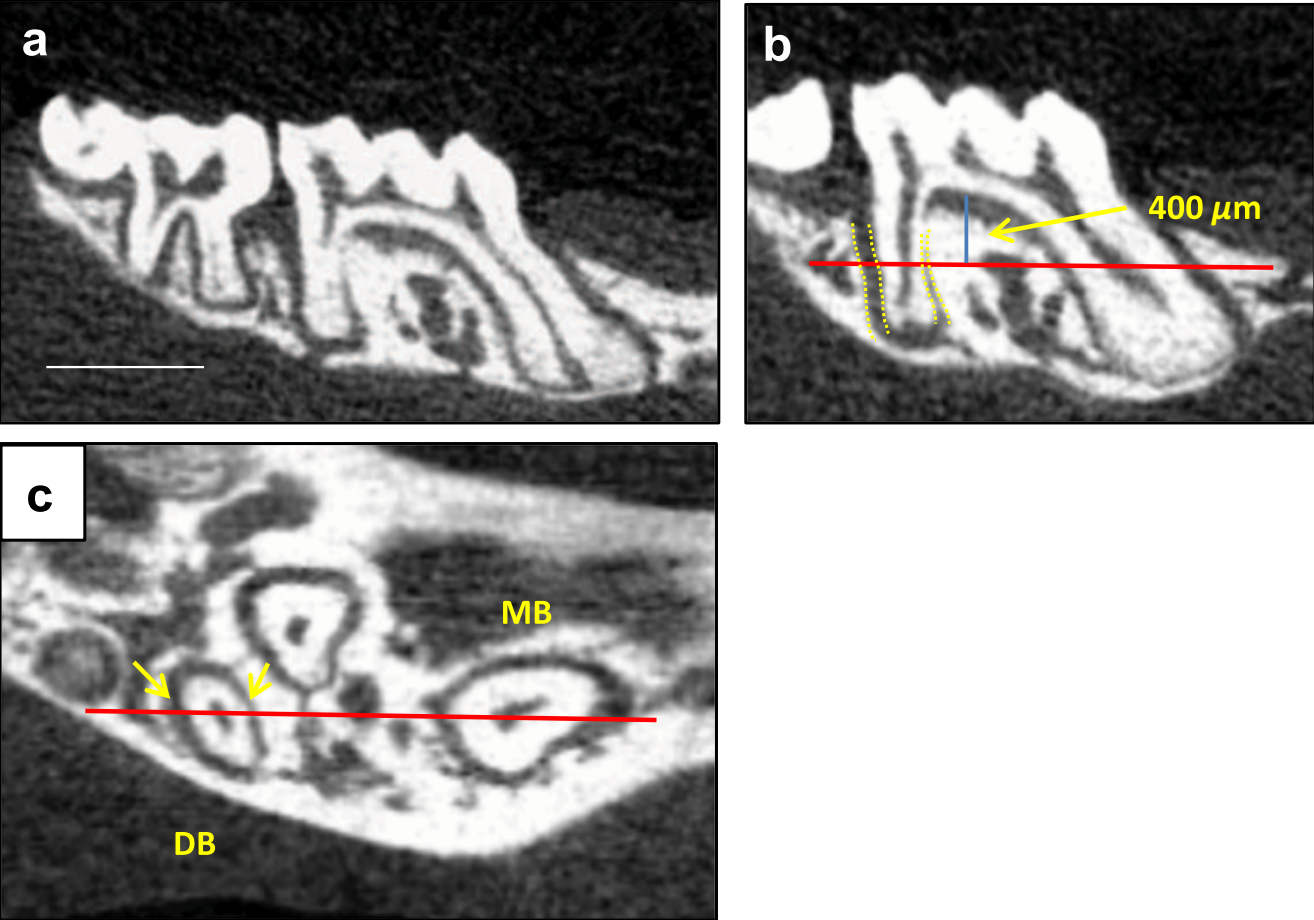
Orthodontic tooth movement and PDL width measurements. **a** Orthodontic tooth movement was quantified by measuring the minimum distance between the maxillary first and second molar crowns. Bar = 1 mm. **b** The average PDL width was quantified by measuring the mean PDL space on the distal aspect of distobuccal root in sagittal micro-CT images. **c** The mid-root PDL width was measured on the distal aspect of the distobuccal root in horizontal micro-CT images 400 μm apical to the furcation as shown in **b**. MB, mesiobuccal root; DB, distobuccal root; PDL, periodontal ligament

After euthanasia, the maxillae were harvested and fixed in 10% paraformaldehyde at 4 °C for 24 h. Samples were scanned using a micro-CT (MicroCT35; SCANCO Medical, Bassersdorf, Switzerland) at 55 kVp and 145 µA intensity with an integration time 200 ms. Maxillary molar areas were scanned at a 20 µm isotropic voxel size. After reconstruction, all images were converted to DICOM files and then imported to OsiriX (Pixmeo SARL, Bernex, Switzerland) for analysis. The amount of orthodontic tooth movement (OTM) and the width of PDL mesial to distobuccal root of maxillary first molar were measured in reconstructed micro-CT images (Fig. 6). Orthodontic tooth movement was measured as the closest distance between the most distal point of maxillary right first molar crown and the most mesial point of maxillary right second molar crown in relation to the same measurements for the left side. We measured the average PDL width at sagittal section and the PDL width at the mid-root coronal section. The sagittal section selected for average PDL width measurement was the radiographic image with both buccal root canals of the maxillary first molar most thoroughly visible. The average PDL width was measured from the coronal to the apical portion of the PDL. For the mid-root PDL width the coronal section 400 μm below the topmost point of furcation was selected. OsiriX (Pixmeo SARL, Bernex, Switzerland) and Image Pro Plus (Media Cybernetics, Inc., Rockville, MD) were used for all the 3D image reconstruction and measurement. Micro-CT data were examined by a double blinded examiner and the results were confirmed with a second examiner.

### Histology and TRAP stain

Specimens were decalcified in 10% EDTA for 5 weeks and paraffin-embedded. 4-μm thick sagittal sections were stained for tartrate resistant acid phosphatase (TRAP; Sigma-Aldrich, Saint Louis, MO) and then counterstained with hematoxylin. In order to count the osteoclast number and measure the PDL width, mesial periodontal region of the distobuccal root of the maxillary first molar was used. Osteoclasts were identified as TRAP-positive multinucleated cells on the bone surface under 10× (resolution: 1.12 µm) and 20× objectives (resolution: 0.67 µm). We investigated the number of osteoclasts from the topmost 100 µm below the furcation to the apex to exclude the possible influence from the adjacent inflammation at furcation area. Images of TRAP-stained sections were captured with a Nikon Eclipse 90i microscope (Nikon, Melville, NY) and Nikon DS-Fi1 camera and analyzed using a NIS Elements-AR software (Nikon). The average PDL width and mid-root PDL width were measured as previously described. Image Pro Plus (Media Cybernetics, Inc., Rockville, MD) was used for the PDL width measurement. Data were examined by a double blinded examiner and the results were confirmed with a second examiner.

### Immunofluorescence

Paraffin-embedded, formalin-fixed sample sections were processed for immunofluorescence analyses. Antigen retrieval was performed in 10 mmol•L^−1^ of citric acid, pH 6.0, at 120 °C. Sections were incubated with primary antibody to ß-galactosidase (rabbit, bs-4960R; Bioss Antibodies, Woburn, MA) or RANKL (Goat, sc 7628, Santa Cruz Biotechnology, Inc., Dallas, TX) overnight at 4 °C, as well as the appropriate isotype-matched negative control IgG. Biotinylated secondary antibody (Thermo Fisher Scientific, Waltham, MA) and ABC reagent (Vector Laboratories, Burlingame, CA, USA) were then used. Tyramide signal amplification (Adipogen, San Diego, CA) was also used to enhance the chromogenic signal. Finally, Alexa Fluor 546–conjugated streptavidin (Invitrogen, Carlsbad, CA) and DAPI-containing mounting media (Sigma-Aldrich, St. Louis, MO) were used to visualize the staining. Images were taken at 20× and 40× magnification with a fluorescence microscope (ECLIPSE 90i; Nikon) with the same exposure time for experimental and negative control groups. Image analysis was performed using a NIS Elements-AR software (Nikon). In all experiments capture times were selected so that no immunofluorescence was detected with control IgG.

### Statistics

All data are reported as the mean ± SEM. Non-parametric analysis to compare sample means was carried out using the Mann-Whitney U test. *P* < 0.05 was considered statistically significant. We performed a power analysis assuming a type I error frequency of 5%, power of the statistical test set at 90% (*P* = 0.9, *b* = 0.1) and an effect size of 60% and found that a sample size of 7 was adequate. In addition we checked the previous literature and found typical sample sizes of 5–8 animals per group.^45,55–57^ For our study we had a sample size of 7–8 animals per group for statistical analysis.

## References

[1] Riancho JA, Delgado-Calle J (2011). [Osteoblast-osteoclast interaction mechanisms]. Reumatol. Clin..

[2] Tanaka Y, Nakayamada S, Okada Y (2005). Osteoblasts and osteoclasts in bone remodeling and inflammation. Curr. Drug. Targets Inflamm. Allergy.

[3] Hadjidakis DJ, Androulakis II (2006). Bone remodeling. Ann. N. Y. Acad. Sci..

[4] Murshid SA (2017). The role of osteocytes during experimental orthodontic tooth movement: a review. Arch. Oral. Biol..

[5] Teitelbaum SL (2000). Bone resorption by osteoclasts. Science.

[6] Yasuda H (1998). Osteoclast differentiation factor is a ligand for osteoprotegerin/osteoclastogenesis-inhibitory factor and is identical to TRANCE/RANKL. Proc. Natl Acad. Sci. USA..

[7] Boyle WJ, Simonet WS, Lacey DL (2003). Osteoclast differentiation and activation. Nature.

[8] O'Brien CA (2010). Control of RANKL gene expression. Bone.

[9] Meikle MC (2006). The tissue, cellular, and molecular regulation of orthodontic tooth movement: 100 years after Carl Sandstedt. Eur. J. Orthod..

[10] Wang XJ (2006). Role of TGF beta-mediated inflammation in cutaneous wound healing. J. Investig. Dermatol. Symp. Proc..

[11] Walker JB, Buring SM (2001). NSAID impairment of orthodontic tooth movement. Ann. Pharmacother..

[12] Mabuchi R, Matsuzaka K, Shimono M (2002). Cell proliferation and cell death in periodontal ligaments during orthodontic tooth movement. J. Periodontal Res..

[13] Pavlin D, Gluhak-Heinrich J (2001). Effect of mechanical loading on periodontal cells. Crit. Rev. Oral. Biol. Med..

[14] Krishnan V, Davidovitch Z (2006). Cellular, molecular, and tissue-level reactions to orthodontic force. Am. J. Orthod. Dentofac. Orthop..

[15] Mitchell DL, West JD (1975). Attempted orthodontic movement in the presence of suspected ankylosis. Am. J. Orthod..

[16] Lekic P, McCulloch CA (1996). Periodontal ligament cell population: the central role of fibroblasts in creating a unique tissue. Anat. Rec..

[17] Diercke K (2011). Compression-dependent up-regulation of ephrin-A2 in PDL fibroblasts attenuates osteogenesis. J. Dent. Res..

[18] Chen YJ (2014). Activation of focal adhesion kinase induces extracellular signal-regulated kinase-mediated osteogenesis in tensile force-subjected periodontal ligament fibroblasts but not in osteoblasts. J. Bone Miner. Metab..

[19] Sen S (2015). Compression induces Ephrin-A2 in PDL fibroblasts via c-fos. J. Dent. Res..

[20] Cho A, Haruyama N, Kulkarni AB (2009). Generation of transgenic mice. Curr. Protoc. Cell. Biol..

[21] Elefteriou F, Yang X (2011). Genetic mouse models for bone studies–strengths and limitations. Bone.

[22] Feil S, Valtcheva N, Feil R (2009). Inducible Cre mice. Methods Mol. Biol..

[23] Ouyang Z (2013). Prx1 and 3.2kb Col1a1 promoters target distinct bone cell populations in transgenic mice. Bone.

[24] Rossert J, Eberspaecher H, de Crombrugghe B (1995). Separate cis-acting DNA elements of the mouse pro-alpha 1(I) collagen promoter direct expression of reporter genes to different type I collagen-producing cells in transgenic mice. J. Cell. Biol..

[25] Huang H, Williams RC, Kyrkanides S (2014). Accelerated orthodontic tooth movement: molecular mechanisms. Am. J. Orthod. Dentofac. Orthop..

[26] Olson C (2012). Orthodontic tooth movement causes decreased promoter expression of collagen type 1, bone sialoprotein and alpha-smooth muscle actin in the periodontal ligament. Orthod. Craniofac. Res..

[27] Sokos D, Everts V, de Vries TJ (2015). Role of periodontal ligament fibroblasts in osteoclastogenesis: a review. J. Periodontal Res..

[28] Beertsen W (1975). Migration of fibroblasts in the periodontal ligament of the mouse incisor as revealed by autoradiography. Arch. Oral. Biol..

[29] Cho MI, Garant PR (2000). Development and general structure of the periodontium. Periodontol..

[30] Chiba M, Mitani H (2004). Cytoskeletal changes and the system of regulation of alkaline phosphatase activity in human periodontal ligament cells induced by mechanical stress. Cell Biochem. Funct..

[31] Yamashita Y, Sato M, Noguchi T (1987). Alkaline phosphatase in the periodontal ligament of the rabbit and macaque monkey. Arch. Oral. Biol..

[32] Alves LB (2015). Expression of osteoblastic phenotype in periodontal ligament fibroblasts cultured in three-dimensional collagen gel. J. Appl. Oral. Sci..

[33] Diercke K (2011). Strain-dependent up-regulation of ephrin-B2 protein in periodontal ligament fibroblasts contributes to osteogenesis during tooth movement. J. Biol. Chem..

[34] Choi Y (2001). Osteoclastogenesis is enhanced by activated B cells but suppressed by activated CD8(+) T cells. Eur. J. Immunol..

[35] Harada Y (2006). Effect of adoptive transfer of antigen-specific B cells on periodontal bone resorption. J. Periodontal Res..

[36] Teng YT (2000). Functional human T-cell immunity and osteoprotegerin ligand control alveolar bone destruction in periodontal infection. J. Clin. Invest..

[37] Baker PJ (2001). T-cell contributions to alveolar bone loss in response to oral infection with Porphyromonas gingivalis. Acta Odontol. Scand..

[38] Davidovitch Z (1988). Neurotransmitters, cytokines, and the control of alveolar bone remodeling in orthodontics. Dent. Clin. North. Am..

[39] Li Y (2011). Expression of osteoclastogenesis inducers in a tissue model of periodontal ligament under compression. J. Dent. Res..

[40] Nishijima Y (2006). Levels of RANKL and OPG in gingival crevicular fluid during orthodontic tooth movement and effect of compression force on releases from periodontal ligament cells in vitro. Orthod. Craniofac. Res..

[41] Kanzaki H (2006). Local RANKL gene transfer to the periodontal tissue accelerates orthodontic tooth movement. Gene Ther..

[42] Kanzaki H (2004). Local OPG gene transfer to periodontal tissue inhibits orthodontic tooth movement. J. Dent. Res..

[43] Ren Y (2007). Cytokine profiles in crevicular fluid during orthodontic tooth movement of short and long durations. J. Periodontol..

[44] Li J (2015). Altered distribution of HMGB1 in the periodontal ligament of periostin-deficient mice subjected to Waldo's orthodontic tooth movement. J. Mol. Histol..

[45] Taddei SR (2012). Experimental model of tooth movement in mice: a standardized protocol for studying bone remodeling under compression and tensile strains. J. Biomech..

[46] Yadav S (2016). The effect of low-frequency mechanical vibration on retention in an orthodontic relapse model. Eur. J. Orthod..

[47] Yamaguchi M (2009). RANK/RANKL/OPG during orthodontic tooth movement. Orthod. Craniofac. Res..

[48] Hemingway F (2011). RANKL-independent human osteoclast formation with APRIL, BAFF, NGF, IGF I and IGF II. Bone.

[49] Kim HR (2014). Reciprocal activation of CD4+ T cells and synovial fibroblasts by stromal cell-derived factor 1 promotes RANKL expression and osteoclastogenesis in rheumatoid arthritis. Arthritis Rheumatol..

[50] Kamiya N (2008). Disruption of BMP signaling in osteoblasts through type IA receptor (BMPRIA) increases bone mass. J. Bone Miner. Res..

[51] Xiong J (2011). Matrix-embedded cells control osteoclast formation. Nat. Med..

[52] Anastassiadis K (2010). A practical summary of site-specific recombination, conditional mutagenesis, and tamoxifen induction of CreERT2. Methods Enzymol..

[53] Vasioukhin V (1999). The magical touch: genome targeting in epidermal stem cells induced by tamoxifen application to mouse skin. Proc. Natl Acad. Sci. USA..

[54] Yadav S (2016). The effect of mechanical vibration on orthodontically induced root resorption. Angle Orthod..

[55] Andrade I (2007). The role of tumor necrosis factor receptor type 1 in orthodontic tooth movement. J. Dent. Res..

[56] Andrade I (2009). CCR5 down-regulates osteoclast function in orthodontic tooth movement. J. Dent. Res..

[57] Shi J (2017). Antibiotic administration alleviates the aggravating effect of orthodontic force on ligature-induced experimental periodontitis bone loss in mice. J. Periodontal Res..

